# EHMTI-0058. Association between Ala379Val polymorphism of lipoprotein-associated phospholipase A2 and migraine without aura in an Iranian population

**DOI:** 10.1186/1129-2377-15-S1-B41

**Published:** 2014-09-18

**Authors:** F Haghdoost, M Gharzi, F Faez, E Hosseinzadeh, A Zandifar, S Zandifar, SH Javanmard

**Affiliations:** 1 Medical Student Research Center, Isfahan University of Medical Sciences, Isfahan, Iran; 2 Pharmacy Student Research Center, Isfahan University of Medical Sciences, Isfahan, Iran; 3 Physiology Research Center, Isfahan University of Medical Sciences, Isfahan, Iran

## Introduction

Migraine is a common neurovascular disorder with multifactorial and polygenic inheritance.

## Aims

The aim of this study was to investigate the association of migraine without aura and Ala379Val polymorphism of lipoprotein-associated phospholipase A2 (Lp- PLA2) gene in an Iranian population.

## Methods

In this study 103 migraine patients and 100 healthy controls were enrolled. DNA samples were extracted and the Ala379Val polymorphism of Lp-PLA2 gene was investigated. To assess severity of headache, patients filled out the HIT-6 and MIGSEV questionnaires.

## Results

Allele V had significantly lower frequency in case group than control subjects (P=0.00, OR=0.25, CI=0.15 - 0.40). The frequency of migraine patients that were carrier of V allele (V/V and A/V) was statistically significant lower than control group (P=0.003, OR=2.39, CI: 1.35 - 4.23). There was no significant difference of alleles frequency between three grades of MIGSEV (P=0.316). Also total HIT-6 score was not significantly different between different genotypes (0.466).

## Conclusion

In conclusion our results showed that Ala379Val gene polymorphism of Lp-PLA2 is associated with lower risk of migraine but not with severity of headaches.

No conflict of interest.

